# Muscle Biopsy Findings in Combination With Myositis‐Specific Autoantibodies Aid Prediction of Outcomes in Juvenile Dermatomyositis

**DOI:** 10.1002/art.39753

**Published:** 2016-10-09

**Authors:** Claire T. Deakin, Shireena A. Yasin, Stefania Simou, Katie A. Arnold, Sarah L. Tansley, Zoe E. Betteridge, Neil J. McHugh, Hemlata Varsani, Janice L. Holton, Thomas S. Jacques, Clarissa A. Pilkington, Kiran Nistala, Lucy R. Wedderburn, on behalf of the UK Juvenile Dermatomyositis Research Group, Kate Armon, Joe Ellis‐Gage, Holly Roper, Vanja Briggs, Joanna Watts, Liza McCann, Ian Roberts, Eileen Baildam, Louise Hanna, Olivia Lloyd, Susan Wadeson, Phil Riley, Ann McGovern, Clive Ryder, Janis Scott, Beverley Thomas, Taunton Southwood, Eslam Al‐Abadi, Sue Wyatt, Gillian Jackson, Tania Amin, Mark Wood, Vanessa VanRooyen, Deborah Burton, Joyce Davidson, Janet Gardner‐Medwin, Neil Martin, Sue Ferguson, Liz Waxman, Michael Browne, Mark Friswell, Alison Swift, Sharmila Jandial, Vicky Stevenson, Debbie Wade, Ethan Sen, Eve Smith, Lisa Qiao, Stuart Watson, Claire Duong, Helen Venning, Rangaraj Satyapal, Elizabeth Stretton, Mary Jordan, Ellen Mosley, Anna Frost, Lindsay Crate, Kishore Warrier, Stefanie Stafford, Nathan Hasson, Sue Maillard, Elizabeth Halkon, Virginia Brown, Audrey Juggins, Sally Smith, Sian Lunt, Elli Enayat, Laura Kassoumeri, Laura Beard, Yvonne Glackin, Beverley Almeida, Raquel Marques, Stefanie Dowle, Charis Papadopoulou, Kevin Murray, John Ioannou, Linda Suffield, Muthana Al‐Obaidi, Helen Lee, Sam Leach, Helen Smith, Anne‐Marie McMahon, Heather Chisem, Ruth Kingshott, Nick Wilkinson, Emma Inness, Eunice Kendall, David Mayers, Ruth Etherton, Kathryn Bailey, Jacqui Clinch, Natalie Fineman, Helen Pluess‐Hall, Lindsay Vallance, Louise Akeroyd, Alice Leahy, Amy Collier, Rebecca Cutts, Hans De Graaf, Brian Davidson, Sarah Hartfree, Danny Pratt

**Affiliations:** ^1^ University College LondonLondonUK; ^2^ University of BathBathUK; ^3^ Great Ormond Street Hospital for Children NHS Foundation TrustLondonUK; ^4^ Great Ormond Street Hospital for Children NHS Foundation Trust and University College LondonLondonUK; ^5^ University College London, Great Ormond Street Hospital for Children NHS Foundation Trust, and University College London HospitalsLondonUK

## Abstract

**Objective:**

Juvenile dermatomyositis (DM) is a rare and severe autoimmune condition characterized by rash and proximal muscle weakness. While some patients respond to standard treatment, others do not. This study was carried out to investigate whether histopathologic findings and myositis‐specific autoantibodies (MSAs) have prognostic significance in juvenile DM.

**Methods:**

Muscle biopsy samples (n = 101) from patients in the UK Juvenile Dermatomyositis Cohort and Biomarker Study were stained, analyzed, and scored for severity of histopathologic features. In addition, autoantibodies were measured in the serum or plasma of patients (n = 90) and longitudinal clinical data were collected (median duration of follow‐up 4.9 years). Long‐term treatment status (on or off medication over time) was modeled using generalized estimating equations.

**Results:**

Muscle biopsy scores differed according to MSA subgroup. When the effects of MSA subgroup were accounted for, increased severity of muscle histopathologic features was predictive of an increased risk of remaining on treatment over time: for the global pathology score (histopathologist's visual analog scale [hVAS] score), 1.48‐fold higher odds (95% confidence interval [95% CI] 1.12–1.96; *P* = 0.0058), and for the total biopsy score (determined with the standardized score tool), 1.10‐fold higher odds (95% CI 1.01–1.21; *P* = 0.038). A protective effect was identified in patients with anti–Mi‐2 autoantibodies, in whom the odds of remaining on treatment were 7.06‐fold lower (95% CI 1.41–35.36; *P* = 0.018) despite muscle biopsy scores indicating more severe disease. In patients with anti–nuclear matrix protein 2 autoantibodies, anti–transcription intermediary factor 1γ autoantibodies, or no detectable autoantibody, increased histopathologic severity alone, without adjustment for the effect of MSA subtype, was predictive of the risk of remaining on treatment: for the hVAS global pathology score, 1.61‐fold higher odds (95% CI 1.16–2.22; *P* = 0.004), and for the total biopsy score, 1.13‐fold higher odds (95% CI 1.03–1.24; *P* = 0.013).

**Conclusion:**

Histopathologic severity, in combination with MSA subtype, is predictive of the risk of remaining on treatment in patients with juvenile DM and may be useful for discussing probable treatment length with parents and patients. Understanding these associations may identify patients at greater risk of severe disease.

Accurate prediction of outcomes is a common problem in rare diseases. For many rare diseases, including juvenile myositis, patients and clinicians have an unmet need with regard to the capacity to predict poor outcomes. A further challenge in the study of rare autoimmune diseases is that disease mechanisms may be unknown, which renders biomarker research difficult. Juvenile dermatomyositis (DM) is an example of a rare disease in which the disease pathogenesis is only partially understood. A chronic autoimmune condition of childhood, juvenile DM is typically characterized by proximal muscle weakness, elevated levels of muscle enzymes in the serum, and rashes such as heliotrope rash and Gottron's papules 1. Other clinical features that may contribute to major morbidity include calcinosis, ulceration, treatment‐resistant rash, and involvement of the gut, lungs, and brain. While some patients achieve disease remission following a regimen of standard therapies, others fail to respond. In a recent long‐term study of outcomes in 59 adults who had previously been diagnosed as having juvenile DM, 51% still had active disease after a median follow‐up of 16 years 2. At present, early biomarkers of disease that are associated with long‐term outcomes have not been identified.

To facilitate research into the biologic mechanisms, biomarkers, and disease outcomes in juvenile DM, the UK Juvenile Dermatomyositis Cohort and Biomarker Study (JDCBS) (n = 506 patients at the time of this analysis) was established to collect serial clinical data and biospecimens 3. Such studies open the potential for the classification of rare diseases into subtypes that are defined by biomarkers and that are associated with predictable outcomes, thus allowing investigation of disease mechanisms that might drive these subtypes. Biomarker research may eventually enable development of therapies directed against more relevant targets for particular subtypes, ultimately leading to better clinical outcomes. Myositis‐specific autoantibodies (MSAs) have been identified in both adult‐onset DM and juvenile DM, and include anti–Mi‐2, anti–melanoma differentiation–associated gene 5 (anti‐MDA5), anti–transcription intermediary factor 1γ (anti–TIF‐1γ, p155/140), and anti–nuclear matrix protein 2 (anti–NXP‐2, p140; also identified as the anti‐MJ autoantibody). Associations between MSAs and certain clinical features of juvenile DM have been demonstrated, suggesting that these autoantibodies may be useful biomarkers 4, 5, 6, 7, 8, 9, 10, 11. However, little is known about the biologic mechanisms underlying different MSA subtypes or how they relate to long‐term prognosis.

We have previously developed and validated a standardized score tool to quantify abnormalities in muscle tissue obtained by biopsy from patients with juvenile DM 12, 13. Use of immunohistochemistry as a means of predicting prognosis and informing treatment is well‐established in more prevalent diseases such as malignancy. Herein, we applied the standardized juvenile DM score tool to muscle biopsy samples from a large cohort of patients with juvenile DM (n = 101) and tested the hypothesis that early histopathologic score data contain information predictive of long‐term treatment status (on or off medication over time).

## PATIENTS AND METHODS

### Patients, biopsy material, and clinical data

Pediatric patients with definite or probable juvenile DM 14 were recruited to the UK JDCBS (n = 506). Written informed parental consent and age‐appropriate assent were obtained from participants prior to inclusion in the study. This research was approved by the UK Northern & Yorkshire Medical Research and Ethics Committee. Members of the UK Juvenile Dermatomyositis Research Group (JDRG) are listed in Appendix A. At the time of this study, muscle biopsy samples from the JDCBS with tissue of sufficient quantity and quality (n = 101) were analyzed.

All tissue samples were obtained by open quadriceps biopsy, with the patient placed under general anesthesia. Most of these patients (94.1%) were treated at Great Ormond Street Hospital for Children (GOSH), a major referral center, where the policy is to perform routine biopsy at the time of diagnosis in patients with juvenile DM. Consequently, a wide range of histopathologic severities, ranging from mild to moderate levels of severity, is represented in these biopsy samples (see Supplementary Figures 1A and B, available on the *Arthritis & Rheumatology* web site at http://onlinelibrary.wiley.com/doi/10.1002/art.39753/abstract). Although the distribution of disease severity scores at diagnosis was more skewed toward increased severity in those in whom a muscle biopsy was performed than in those in whom it was not performed (see Supplementary Table 1 and Supplementary Figures 1C and D, http://onlinelibrary.wiley.com/doi/10.1002/art.39753/abstract), the patients who did not undergo biopsy were also more likely to have missing data at diagnosis, and therefore these data are difficult to interpret.

Clinical data collected at the time of diagnosis and time of muscle biopsy included the physician's global assessment of disease activity (score range 0–10, with low scores indicating minimal disease), Manual Muscle Testing in 8 muscles (score range 0–80, with high scores indicating no muscle weakness) 15, Childhood Myositis Assessment Scale (score range 0–52, with high scores indicating no weakness) 16, and serum creatine kinase levels (in units/liter). Treatments received by patients were also recorded at each clinic visit. At the time of diagnosis, all patients received methotrexate, and the majority received concomitant steroids, in accordance with international protocols 17. In patients in whom disease was unresponsive to treatment with methotrexate, other disease‐modifying antirheumatic drugs were administered, including azathioprine, hydroxychloroquine, intravenous immunoglobulin, and cyclophosphamide. For patients who continued to have refractory disease, anti–tumor necrosis factor biologic agents (infliximab or adalimumab) were administered. None of the analyzed patients were treated with cyclosporin A.

### Histologic analyses and scoring of biopsy samples

Histologic staining and analysis and scoring of biopsy samples were conducted as described previously, using the validated juvenile DM biopsy score tool to calculate a total biopsy score 12, 13. The histopathologist's visual analog scale (hVAS) global pathology score provides a global assessment of the severity of histopathologic features in muscle biopsy tissue. Values for the total biopsy score (which includes assessment in 4 domains) range 0–27 and those for the hVAS score range 0–10, with higher scores indicating greater severity.

All histologic assessments and scoring were performed by a single observer (SAY), who was blinded with regard to the autoantibody status of each patient with juvenile DM and who was trained by 2 highly qualified consultant neuropathologists (TSJ and JLH) who are experienced specialists in the field and were involved in the development and validation of the juvenile DM biopsy score tool 12, 13. To ensure reliability, scores for the initial 9 muscle biopsy samples evaluated were cross‐compared to, first, the scores from the 2 trainers, and second, those generated by an international panel during the validation of the score tool 13. The intraclass correlation coefficient for the hVAS score from the observer and those from the international panel in the 9 samples was 0.80 (95% confidence interval [95% CI] 0.62–0.95), indicating high levels of agreement.

### Autoantibody screening

Serum or plasma from patients with juvenile DM were screened for autoantibodies using immunoprecipitation analyses, as described previously 5, 9, 10, 11. Specificity for anti–NXP‐2 or anti‐MDA5 autoantibodies, visualized as a 140‐kd band, was determined by enzyme‐linked immunosorbent assay, as described previously 5, 11. Since recent studies in the literature have identified important associations between the clinical features of juvenile DM and the presence of MSAs 4, 5, 6, 7, 8, 9, 10, 11, and relatively fewer patients with myositis‐associated autoantibodies (MAAs) were present in the biopsy cohort (with low numbers in individual groups), we elected to focus on the patient groups in whom the frequencies of MSAs, i.e., anti–TIF‐1γ, anti–NXP‐2, anti‐MDA5, and anti–Mi‐2, were sufficient for these analyses. Patients with MAAs or unidentified bands were excluded during the statistical analyses of associations with muscle biopsy scores and associations between muscle biopsy scores, MSA subtypes, and long‐term outcomes. Patients with no detectable autoantibody were included.

### Statistical analysis

Correlations between the total biopsy score and hVAS score were analyzed using Spearman's rank correlation coefficients in R, version 3.2.1 18. A factorial analysis of variance (ANOVA) was conducted using the nonparametric Kruskal‐Wallis test in R, to identify significant main effects of MSA subgroups on biopsy scores. Post hoc comparisons to identify pairs of MSA subgroups whose biopsy scores significantly differed from each other were performed using Dunn's test in the R package dunn.test, with *P* values adjusted using the Bonferroni method 19. For each medication, the distribution of whether that drug was ever received by patients across each MSA subgroup was analyzed using Fisher's exact test in R, with *P* values adjusted using the Bonferroni method.

A longitudinal modeling approach, which could include all available time points for each patient, was adopted for the analysis of the treatment status outcome, in order to make maximal use of the available serial clinical data. A longitudinal approach was preferred over a cross‐sectional approach, which is limited to arbitrarily selected time points of interest and ignores any other time points. Recurrent event analysis was preferred over time‐to‐event analysis, which is limited to time points up to the first time at which patients come off treatment and ignores subsequent time points when patients may come on treatment again.

Longitudinal modeling of long‐term treatment status was performed using generalized estimating equations (GEEs), a longitudinal method for analyzing recurrent events that provides more conservative estimates for modeling binary outcomes than mixed‐effects models 20, 21, 22. GEE models were fitted using the R package geepack and an autoregressive correlation structure 23. Date of diagnosis was considered the baseline time point. Time from diagnosis was used as the time variable, which ensured that adjustment was made for the effects of treatment duration. The no detectable autoantibody group (n = 20) was the reference category for the MSA variable, to enable more precise estimates, since this group had the most patients. Although the patients who underwent muscle biopsy are predominantly an inception cohort, a mixture of patients with incident disease and those with prevalent disease was recruited when the JDCBS was first started. For this reason, time from disease onset to diagnosis and time from diagnosis to biopsy were considered as potential confounders in longitudinal modeling. Since time from disease onset to diagnosis and time from diagnosis to biopsy were both found to have significant effects, these confounders were retained as covariates in subsequent analyses. Thus, all parameter estimates are adjusted for the effects of time from disease onset to diagnosis and time from diagnosis to biopsy.

Additional potentially confounding variables were evaluated, and included sex, whether steroids had been received before biopsy, and ever treatment with cyclophosphamide, but none of these had a significant effect in the model, and therefore these variables were not retained. Estimates of odds ratios (ORs) are presented as odds of being on treatment, with 95% CIs. Since ORs below 1 can be difficult to interpret, an OR of <1 is also presented as the odds of being off treatment.

Parameter estimates from the GEE models were used to formulate an equation to calculate the odds and, thus, the predicted probability of being off treatment. To enable predicted probability to be plotted as a function of the hVAS score or total biopsy score, a fixed time point of 5 years postdiagnosis was used. Median values for the time from onset to diagnosis and time from diagnosis to biopsy were used for confounding variables. Plots were generated using a customized R function and the base plotting system.

Bivariate, univariate, and null GEE models were compared using ANOVA for comparison of nested models, and the R package MuMIn was used for calculation of the quasi–Akaike's information criterion (QIC) and the proportion of weighting for the preferred model using the function “model.sel()” 24. ANOVA uses a chi‐square distribution to test the likelihood ratios of the models being compared. QIC is a measure of the relative quality of a GEE model, with lower values indicating an improved fit. For the comparisons between the models, the QIC values, proportion of weighting calculated by model.sel(), chi‐square statistics, and *P* values are reported.

For longitudinal modeling, *P* values less than 0.05 were considered statistically significant. Summary statistics are presented as the median and interquartile range for numeric variables, and as counts and percentages for categorical variables. The 95% CIs are presented for all estimated parameters. Figures depicting correlations and distributions of biopsy scores and forest plot depictions of the ORs were generated using GraphPad Prism (version 5.01; GraphPad Software).

## RESULTS

### Demographic, clinical, and serologic features of the muscle biopsy cohort

The 101 patients included in this analysis were predominantly female and white, and their clinical features indicated a range of disease severities (Table 1). The MSAs analyzed in this study were detected in 58.9% of the 90 patients screened, including anti–TIF‐1γ in 20.0% of patients, anti–NXP‐2 in 16.7% of patients, anti‐MDA5 in 12.2% of patients, and anti–Mi‐2 in 5.6% of patients. MAAs were detected in 10.0% of patients, while unidentifiable bands were detected in 8.9% of patients and no autoantibodies were detected in 22.2% of patients.

**Table 1:** Demographic, clinical, and serologic features of the cohort of patients who underwent muscle biopsy (n = 101)^a^

Sex, no. (%)	
Male	33 (32.7)
Female	68 (67.3)
Ethnicity, no. (%)	
White	72 (71.3)
Black	12 (11.9)
South Asian	8 (7.9)
Other	9 (8.9)
Clinical features at biopsy, median (IQR)^b^	
Age at disease onset, years	6.1 (3.9–9.3)
Physician's global assessment of disease activity (scale 0–10)	4.1 (2.0–7.0)
MMT‐8 (scale 0–80)	55.0 (40.0–71.5)
CMAS (scale 0–52)	29 (18.75–45)
Creatine kinase, units/liter	213 (55–1,019)
Clinical features at biopsy, median (IQR)	
Time from disease onset to diagnosis, months	2.6 (1.5–7.5)
Time from diagnosis to biopsy, months	0.72 (0.39–0.92)
Biopsy performed >1 month after diagnosis, no. (%)	17 (16.8)
Taking steroids at biopsy, no. (%)^c^	12 (12.2)
Myositis‐specific autoantibodies, no. (%)^d^	53 (58.9)
Anti–TIF‐1γ	18 (20.0)
Anti–NXP‐2	15 (16.7)
Anti‐MDA5	11 (12.2)
Anti–Mi‐2	5 (5.6)
Anti‐SRP	2 (2.2)
Anti–PL‐7	1 (1.1)
Anti‐SAE	1 (1.1)
Myositis‐associated autoantibodies, no. (%)	9 (10.0)
Anti–PM‐Scl	6 (6.7)
Anti–U1 RNP	2 (2.2)
Anti‐topo	1 (1.1)
Unidentified autoantibodies, no. (%)	8 (8.9)
No detectable autoantibodies, no. (%)	20 (22.2)

a IQR = interquartile range; anti–TIF‐1γ = anti–transcription intermediary factor 1γ; anti–NXP‐2 = anti–nuclear matrix protein 2; anti–MDA5 = anti–melanoma differentiation–associated gene 5; anti–SRP = anti–signal recognition particle; anti–PL‐7 = anti–threonyl–transfer RNA synthetase; anti‐SAE = anti–small ubiquitin‐like modifier activating enzyme; anti‐topo = antitopoisomerase.

b Clinical features were missing for some patients, as follows: for physician's global assessment of disease activity, n = 11; for Manual Muscle Testing in 8 muscles (MMT‐8), n = 42; for Childhood Myositis Assessment Scale (CMAS), n = 17; for creatine kinase levels, n = 30.

c Steroid use not recorded at the time of biopsy for 3 individuals (3.0%).

d Autoantibodies were screened in the serum or plasma of 90 patients who underwent muscle biopsy. Percentages reflect the number of patients with a given antibody as a proportion of the total patients tested.

### Differential distribution of muscle biopsy scores according to autoantibody status

**Figure 1:**
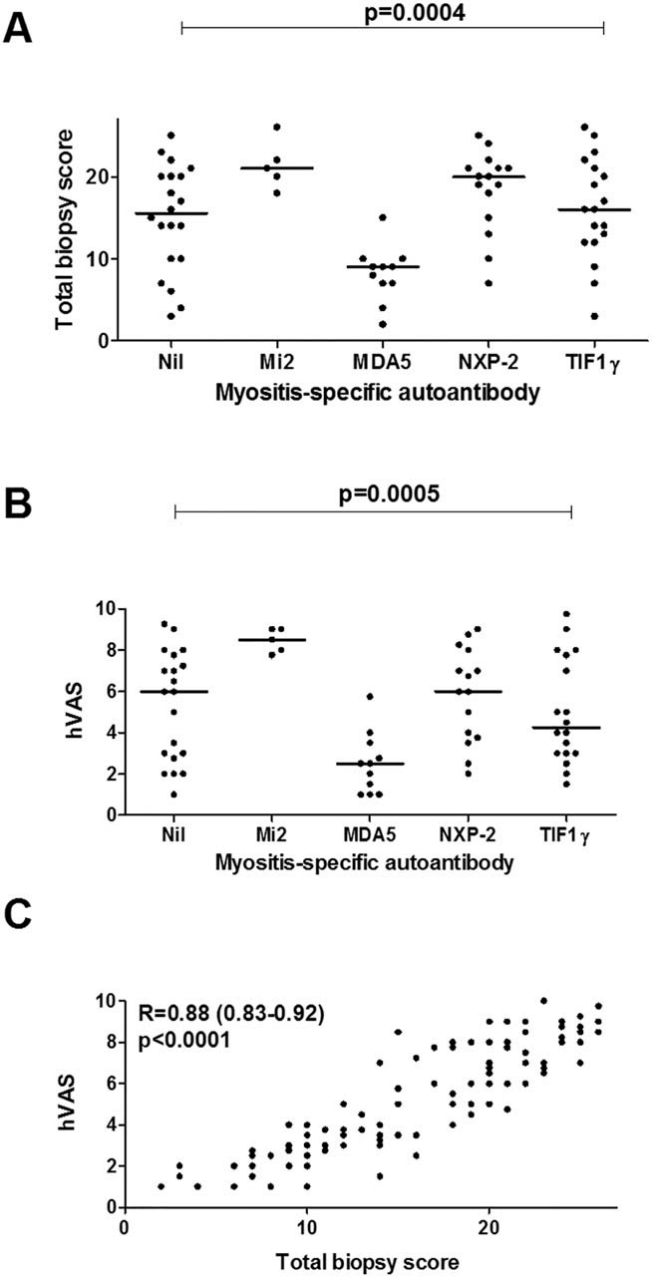
Distributions and correlations of total biopsy scores and histopathologist's visual analog scale (hVAS) global pathology scores in patients with juvenile dermatomyositis. **A** and **B,** The distribution of total biopsy scores **(A)** and hVAS scores **(B)** was determined across subgroups of patients with myositis‐specific antibodies (MSAs) or no detectable autoantibody (nil) (n = 69). Factorial analysis of variance using the Kruskal‐Wallis test was performed to analyze the distribution of these scores. There was a significant main effect of MSA subtype on the hVAS score (χ^2^ [df4] = 20.0, *P* = 0.0005; n = 69): for anti–melanoma differentiation–associated gene 5 (anti‐MDA5) vs. anti–Mi‐2, *P* = 0.0001; for anti‐MDA5 vs. anti–nuclear matrix protein 2 (anti–NXP‐2), *P* = 0.007; for anti‐MDA5 vs. anti–transcription intermediary factor 1γ (anti–TIF‐1γ), *P* = 0.04; for anti‐MDA5 vs. no detectable autoantibody, *P* = 0.03. There was also a significant main effect of MSA subtype on the total biopsy score (χ^2^ [df4] = 20.4, *P* = 0.0004; n = 69): for anti‐MDA5 vs. anti–Mi‐2, *P* = 0.0009; for anti‐MDA5 vs. anti–NXP‐2, *P* = 0.0006; for anti‐MDA5 vs. anti–TIF‐1γ, *P* = 0.01; for anti‐MDA5 vs. no detectable autoantibody, *P* = 0.04. Symbols indicate individual patients; bars show the median. **C,** Correlation of total biopsy scores and hVAS scores was determined by Spearman's rank correlation analysis (n = 101) (expressed as R values with 95% confidence intervals).

The biopsy hVAS global pathology scores and total biopsy scores were highly correlated (R = 0.88, *P* < 0.0001) (Figure 1C), which is indicative of the internal consistency of the standardized tool. Biopsy hVAS scores and total biopsy scores included both low and high scores and were not skewed toward either the more severe levels or milder levels of disease (see Supplementary Figures 1A and B).

Interestingly, there were clear differences in the distribution of both the hVAS scores and total biopsy scores between the major MSA subgroups and patients with no detectable autoantibodies (for the total biopsy scores, *P* = 0.0004; for the hVAS scores, *P* = 0.0005) (Figures 1A and B). Typically, patients with anti–Mi‐2 autoantibodies displayed severe levels of disease, while those with anti‐MDA5 autoantibodies displayed mild disease. Patients with anti‐MDA5 autoantibodies had significantly lower hVAS and total biopsy scores compared to all other groups. Variable levels of histopathologic severity were observed in patients with anti–NXP‐2, those with anti–TIF‐1γ, and those with no detectable autoantibody.

### Association of muscle biopsy histopathologic scores with long‐term treatment status, and influence of autoantibody status

We next investigated whether the MSA subgroups and histopathologic severity were associated with the long‐term outcome of continued treatment over time. Long‐term treatment status (on or off medication over time) was selected as an outcome of clinical importance to both patients and clinicians. Medications included the immunosuppressive, chemotherapeutic, and biologic agents detailed in Patients and Methods, and were not significantly differently distributed across the MSA subgroups (see Supplementary Table 2, available on the *Arthritis & Rheumatology* web site at http://onlinelibrary.wiley.com/doi/10.1002/art.39753/abstract).

**Figure 2:**
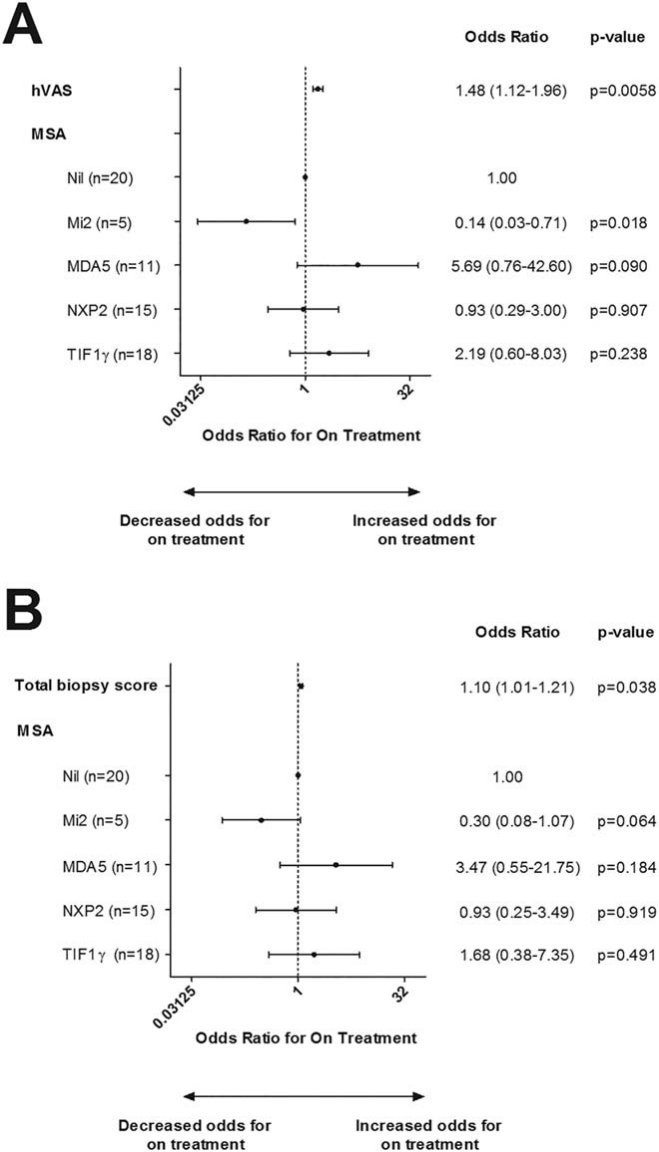
Longitudinal generalized estimating equations (GEE) modeling of treatment status over time according to MSA subgroups and hVAS global muscle pathology scores or total biopsy scores. Forest plots depict odds ratios with 95% confidence intervals for being on treatment, estimated using GEE models fitted with MSA subgroups and either hVAS scores **(A)** or total biopsy scores **(B)** as predictors. The no detectable autoantibody group (nil) was used as the reference category. See Figure 1 for other definitions.

In the GEE models fitted with the MSA subgroups and either the hVAS global pathology score or total biopsy score as covariates, both the hVAS score and the total biopsy score had a significant effect on long‐term treatment status (Figures 2A and B). In the model fitted with the hVAS score and MSA subgroup as covariates, a 1‐unit increase in the hVAS score was associated with 1.48‐fold higher odds (95% CI 1.12–1.96; *P* = 0.0058) of being on treatment over time (Figure 2A). The overall pattern of the GEE model fitted with both MSA subgroup and the total biopsy score as covariates was similar to the model fitted with both MSA subgroup and the hVAS score, although the magnitude of the effect sizes and statistical significance were smaller (Figure 2B). A 1‐unit increase in the total biopsy score was associated with 1.10‐fold higher odds (95% CI 1.01–1.21; *P* = 0.038) of being on treatment over time.

### Association of anti–Mi‐2 autoantibodies with higher odds of coming off treatment

Interestingly, the anti–Mi‐2 antibody appeared to have a protective effect in patients with juvenile DM, with these patients having 7.06‐fold lower odds (95% CI 1.41–35.36; *P* = 0.018) of remaining on treatment over time. This finding was counterintuitive, since these patients had more severe muscle disease (as indicated by the total biopsy and hVAS global pathology scores [Figures 1A and B]). However, this estimate had wide 95% CIs, and therefore the findings warrant cautious interpretation. In the model fitted with the total biopsy score as a covariate, anti–Mi‐2 had a protective effect that was borderline insignificant. In contrast to those with anti–Mi‐2, patients with anti‐MDA5 antibodies displayed a nonsignificant trend toward higher odds of remaining on treatment over time, despite having less severe muscle disease at the time of biopsy.

### Capacity of muscle histopathologic severity or autoantibody subgroup alone to predict prognosis

To examine whether the biopsy hVAS global pathology score, total biopsy score, or MSA subgroup alone could be predictive of long‐term treatment status, univariate GEE models were fitted (Table 2). In these univariate models, neither the hVAS score nor the total biopsy score nor any of the MSA subgroups alone had significant effects, even though significant effects of these measures were identified in the bivariate models in which both muscle histopathologic scores and MSA subtypes were included.

**Table 2:** Summary of alternative generalized estimating equations models^a^

Model, predictor variable	Odds ratio (95% CI)	*P*
Univariate models (n = 69) hVAS global pathology score	1.10 (0.92–1.31)	0.28
Total biopsy score	1.03 (0.96–1.10)	0.43
MSAs		
No detectable autoantibodies (n = 20)	1.00	
Anti‐MDA5 (n = 11)	1.69 (0.38–7.60)	0.50
Anti–NXP‐2 (n = 16)	1.61 (0.41–6.36)	0.50
Anti–TIF‐1γ (n = 17)	2.06 (0.46–9.28)	0.35
Anti–Mi‐2 (n = 5)	0.68 (0.24–1.90)	0.46
Bivariate model (n = 44)†		
Physician's global assessment of disease activity at diagnosis (n = 44)	1.27 (0.92–1.76)	0.15
MSAs		
No detectable autoantibodies (n = 10)	1.00	
Anti‐MDA5 (n = 9)	1.56 (0.22–11.00)	0.65
Anti–NXP‐2 (n = 9)	0.44 (0.08–2.55)	0.36
Anti–TIF‐1γ (n = 12)	1.51 (0.20–11.16)	0.69
Anti–Mi‐2 (n = 4)	0.78 (0.09–7.00)	0.83

a 95% CI = 95% confidence interval; hVAS = histopathologist's visual analog scale; MSAs = myositis‐specific autoantibodies; anti–MDA5 = anti–melanoma differentiation–associated gene 5; anti–NXP‐2 = anti–nuclear matrix protein 2; anti–TIF‐1γ = anti–transcription intermediary factor 1γ.

Scores for the physician's global assessment of disease activity at diagnosis were available for 44 patients.

When the univariate models were compared to the bivariate models, we found that the bivariate models were a better fit for the data (Table 3). Therefore, when all patients with MSAs were assessed, muscle biopsy scores alone or presence of MSAs alone were not predictive of prognosis.

**Table 3:** Summary of model comparisons

			ANOVA^c^	
	QIC^a^	Model selection weight^b^	χ^2^	*P*
Bivariate vs. nested univariate and null, for models fitted with patients with MSAs (n = 69)				
Bivariate (hVAS score and MSAs)				
Alone	315	–	–	–
vs. univariate (hVAS score only)	349	1	10.2 (4)	0.038
vs. univariate (MSAs only)	355	1	7.6 (1)	0.0058
vs. null (time only)	350	1	10.5 (5)	0.063
Bivariate (total biopsy score and MSAs)				
Alone	336	–	–	–
vs. univariate (total biopsy score only)	351	0.999	8.6 (4)	0.073
vs. univariate (MSAs only)	355	1	4.3 (1)	0.038
vs. null (time only)	350	0.999	8.6 (5)	0.13
Bivariate (MSAs and PGA) vs. bivariate (MSAs and either hVAS score or total biopsy score) (n = 44)^d^				
Bivariate (MSAs and PGA)				
Alone	316	–	–	–
vs. bivariate (MSAs and hVAS score)	263	0	–	–
vs. bivariate (MSAs and total biopsy score)	293	0	–	–
Univariate vs. bivariate and null, for models fitted with patients with anti–NXP‐2, patients with anti–TIF‐1γ, and patients with no detectable MSAs (n = 52)^e^				
Univariate (hVAS score)				
Alone	203	–	–	–
vs. bivariate (hVAS and MSAs)	199	0.85	2.0 (2)	0.36
vs. null (time only)	247	1	8.3 (1)	0.004
Univariate (total biopsy score)				
Alone	228	–	–	–
vs. bivariate (total biopsy score and MSAs)	235	0.96	0.7 (2)	0.71
vs. null (time only)	247	1	6.2 (1)	0.013

a Quasi–Akaike's information criterion (QIC) is a measurement of the relative quality of the generalized estimating equations models. Models with lower values indicate a better fit.

b Model selection weight represents the proportion of weight to be given to the bivariate models as compared to their respective nested univariate model or the model with physician's global assessment of disease activity at diagnosis (PGA) and myositis‐specific autoantibody (MSA) subgroup, as compared to the models with biopsy score and MSA subgroup, on a scale of 0–1, when the bivariate, univariate, and null models are compared as indicated. Values of or close to 1 indicate the preferred model.

c Analysis of variance (ANOVA) was used to compare the bivariate model to the nested or null models, with results expressed as the chi‐square value (degrees of freedom). The ANOVA tests for a reduction in residual sum of squares, with *P* values less than 0.05 indicating a significantly improved fit for the data.

d PGA scores at biopsy were available for 44 patients. For the purpose of these model comparisons, the models with MSA subgroup and histopathologist's visual analog scale (hVAS) global pathology score or MSA subgroup and total biopsy score were fitted on the equivalent data set.

e Anti–NXP‐2 = anti–nuclear matrix protein 2; anti–TIF‐1γ = anti–transcription intermediary factor 1γ.

### Identifying muscle biopsy scores as a better prognostic indicator than physician's global assessment of disease activity at diagnosis

We also tested whether substituting the muscle biopsy scores for histopathologic severity with the physician's global assessment of disease activity at diagnosis would result in better prediction of treatment status. The physician's global assessment at diagnosis did not have a statistically significant effect (Table 2), and this model was not a better fit than the models with MSA subgroup and either the hVAS global pathology score or total biopsy score as covariates (Table 3).

### Identifying muscle biopsy scores as a predictor of long‐term treatment status in patients with anti–NXP‐2, patients with anti–TIF‐1γ, and autoantibody‐negative patients

**Figure 3:**
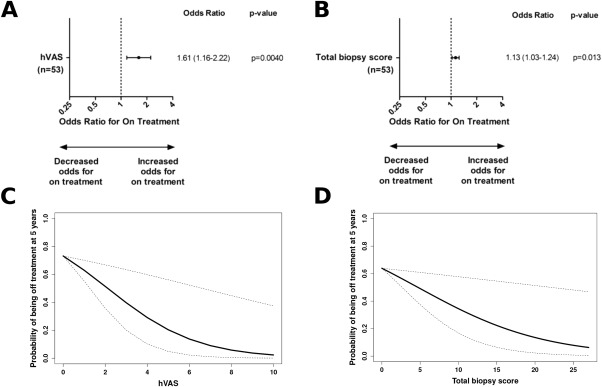
Longitudinal generalized estimating equations (GEE) models of the association between muscle biopsy scores and long‐term treatment status (on or off medication over time) in patients with anti–NXP‐2 autoantibodies, patients with anti–TIF‐1γ autoantibodies, and patients with no detectable autoantibody. **A** and **B,** Forest plots depict odds ratios with 95% confidence intervals for being on treatment, estimated using GEE models fitted with either the hVAS scores **(A)** or the total biopsy scores **(B)** as predictors. **C** and **D,** The predicted probability of being off treatment at 5 years postdiagnosis is plotted as a function of either the hVAS scores **(C)** or the total biopsy scores **(D)**, derived from the GEE models. Dotted lines represent the 95% confidence intervals. The median values for the time from onset to diagnosis (median 0.214 years) and for the time from diagnosis to biopsy (median 0.0602 years) were used in the calculations of predicted probabilities. See Figure 1 for other definitions.

Given the divergent effects of anti–Mi‐2 and anti‐MDA5 in the GEE models fitted with MSAs and muscle biopsy scores (Figure 2), we reasoned that taking out these diametrically opposed groups and removing any potentially overshadowing effects would enable further analysis of the anti–NXP‐2, anti–TIF‐1γ, and autoantibody‐negative subgroups, which were the most prevalent subgroups of patients. In this analysis, muscle biopsy scores alone were associated with long‐term treatment status (Figure 3). A 1‐unit increase in the hVAS global pathology score was associated with 1.61‐fold higher odds (95% CI 1.16–2.22; *P* = 0.004) of remaining on treatment over time (Figure 3A), while a 1‐unit increase in the total biopsy score was associated with 1.13‐fold higher odds (95% CI 1.03–1.24; *P* = 0.013) of remaining on treatment over time (Figure 3B). Inclusion of MSAs as a covariate did not improve the fit (Table 3).

Furthermore, the odds ratio estimates from these univariate models in which just the patients with anti–NXP‐2, patients with anti–TIF‐1γ, and autoantibody‐negative patients were considered were similar to those in the models in which all MSA subgroups were considered and the effects of MSA subgroup were accounted for (Figure 2). This indicates that these sets of models are equivalent, and that there is a need to account for the effects of MSA subgroup when all of the MSA subgroups are considered in the analyses.

Finally, to facilitate interpretation of these models, the predicted probability of being off treatment was plotted as a function of muscle biopsy scores of histopathologic severity at the given time point of 5 years postdiagnosis (Figures 3C and D). These representations show that the predicted probability of being off treatment at 5 years decreases as muscle disease becomes more severe. For example, a patient with anti–NXP‐2 autoantibodies, anti–TIF‐1γ autoantibodies, or no detectable autoantibody and an hVAS global pathology score lower than 2 would have a >50% probability of being off treatment 5 years after the diagnosis. However, if the hVAS score is higher than 8, the estimated probability of being off treatment at 5 years after the diagnosis would be <6%.

## DISCUSSION

The findings of the present study demonstrate that MSA subgroups are linked to the severity of muscle disease in juvenile myositis. Furthermore, we show that MSA subgroups can influence the relationship between muscle biopsy scores and long‐term treatment status in juvenile DM. Such knowledge may assist in the identification of patients who are more likely to respond to treatment, thus distinguishing them from patients who are less likely to respond and may need more aggressive treatment early in the disease.

This is also the first study to identify long‐term clinical patterns in juvenile DM patients with anti–Mi‐2 autoantibodies. It is intriguing that the presence of anti–Mi‐2 was associated with a good prognosis despite being linked to severe muscle disease, and also that there appeared to be an opposite trend in those with anti‐MDA5. It may be that existing immunosuppressive therapies are more effective against the predominant muscle involvement that characterizes the disease in patients with anti–Mi‐2 autoantibodies when compared to that in patients with anti‐MDA5, who have more extramuscular features. For patients with anti–NXP‐2, anti–TIF‐1γ, or no detectable autoantibodies, histopathologic severity alone was predictive of the probability of remaining on treatment over time. Our results suggest that the treatment response is MSA specific, implying that the pathophysiologic features in MSA subgroups are distinct. Therefore, there is a need for further research and development of therapies that would target specific pathways identified as aberrant in these subtypes.

In addition to their usefulness for confirming the diagnosis, our analysis shows that muscle biopsy scores contain important information that, in combination with the MSA status, has prognostic significance. If our findings are replicated using larger patient numbers, performance of muscle biopsy as a routine measure during the diagnostic examination may be justified in patients with juvenile DM. Findings of a recent study in adult patients with DM also suggested that the histopathologic severity varied with MSA subgroups; however, that study did not include an analysis of MSA subgroups and severity of histopathologic features as they related to outcomes 25. In other fields, such as breast cancer and glomerulonephritis, histologic analysis is used to classify a heterogeneous disease into subtypes to inform optimal treatment regimens 26, 27. Such stratified approaches to medication optimization may also be applied to rare heterogeneous diseases such as juvenile DM.

In our analysis, the effect size and statistical significance of the hVAS global pathology score were greater than those of the total biopsy score. Although this global pathology score correlates well with the standardized biopsy score, these 2 parts of the biopsy tool may measure the disease in different ways. The hVAS score has more flexibility and sensitivity to give weight to features that affect histopathologic severity, features that are unaddressed by the specific items within the score tool. Even though it is based on the individual histopathologist's judgment, the hVAS score was found to have high inter‐ and intraobserver reliability during the development and validation of the score tool 12, 13.

Although this study used a large number of biopsy samples from patients with juvenile DM (n = 101), the relatively low numbers of patients within the MSA subgroups limits the precision of the GEE estimates for associations with specific MSA subgroups, resulting in wide confidence intervals. For example, the protective effect identified for anti–Mi‐2 was based on just 5 patients, and the estimate had a wide 95% CI, although the statistical significance of the association nonetheless holds.

Low numbers of patients also restricted our ability to fit more complex models, such as allowing for interactions between MSA subgroups and muscle biopsy scores. Because the numbers of patients in the individual MSA subgroups were low, we consider the most reasonable interpretation of our analysis to be that the histopathologic severity is predictive of long‐term treatment status, and that this effect is influenced by MSA subgroup. This finding is based on all of the patients with MSAs analyzed (n = 69). Ideally, these findings should next be validated in an independent patient cohort, but at present, there are few centers that routinely obtain muscle biopsy samples from patients with juvenile DM, and to our knowledge, this study represents the largest juvenile DM cohort for which biopsy data have been linked to autoantibody status and for which up to 15 years of clinical data have been obtained. As other juvenile DM cohorts with biopsy data are built on, it will be important to use these to validate the present findings. Low patient numbers is a challenge for any study of a rare disease, and the knowledge gained from this study highlights the importance of long‐term biospecimen studies in rare disease cohorts. We also recognize that our findings cannot be extrapolated beyond the MSA subgroups analyzed, and further studies should examine the associations between MAAs and histopathologic severity in greater numbers of patients.

A second limitation of this study is that the treatment status outcome modeled herein was linked only indirectly to muscle biopsy scores. Treatment status was selected as an outcome that would be meaningful to patients and clinicians, and which could be addressed using our data set. It also fluctuates less than the other outcome measures that we have previously considered, such as “clinically insignificant” disease 28, and thus is more amenable to fitting complex longitudinal models. Defining appropriate outcome measures is still an active area of juvenile DM research, and new measures will facilitate research into biomarkers and outcomes. Since not all of the treating clinicians were blinded with regard to the biopsy and MSA results, it is possible that those findings could have influenced the treatment practice, although the relationship between histologic findings, MSAs, and outcomes is still at the research stage, and was not known to clinicians at the time that the treatment choices were made.

Although we sought to include as many biopsy specimens as possible, in practice most of the patients who underwent muscle biopsy were treated at a single center (GOSH) and displayed a full range of disease severity scores at diagnosis. Since a typical overall range of disease severities is represented, our predictive model does accommodate a wide range of mild to severe disease levels. Importantly, a full range of severities of muscle disease was represented in the cohort analyzed for histopathologic features in muscle biopsy samples. However, given that in the UK cohort as a whole, those patients who underwent a muscle biopsy had, on average, more severe disease than those who did not undergo a biopsy, we acknowledge that this skew toward greater disease severity may limit the generalizability of our findings, until more centers can generate further samples that represent the typical distribution of disease severity and can be obtained from patients with known autoantibody status and longitudinal data on outcomes. Nonetheless, our findings are internally valid with respect to the patients from whom biopsy, MSA status, and longitudinal outcomes data are available at present.

In summary, we have shown that histopathologic severity and autoantibody status are correlated with one another, and that muscle biopsy scores, influenced by MSA status, are predictive of the probability of remaining on treatment in patients with juvenile DM. Understanding the link between these early biomarkers of disease and long‐term outcomes may give further insight into different subphenotypes of the disease and lead to more tailored therapies. Our biomarker‐based modeling strategy may well be applied to adult cohorts of patients with inflammatory myositis and may also be a useful approach for the analysis of other rare diseases.

## AUTHOR CONTRIBUTIONS

All authors were involved in drafting the article or revising it critically for important intellectual content, and all authors approved the final version to be published. Dr. Wedderburn had full access to all of the data in the study and takes responsibility for the integrity of the data and the accuracy of the data analysis.

### Study conception and design

Deakin, Holton, Jacques, Pilkington, Nistala, Wedderburn.

### Acquisition of data

Deakin, Yasin, Simou, Arnold, Tansley, Betteridge, Varsani.

### Analysis and interpretation of data

Deakin, Yasin, McHugh, Holton, Jacques, Pilkington, Nistala, Wedderburn.

## Supporting information

Supplementary Figure 1

Supplementary Tables 1

## APPENDIX A MEMBERS OF THE UK JUVENILE DERMATOMYOSITIS RESEARCH GROUP

In addition to some of the authors, members of the UK Juvenile Dermatomyositis Research Group who contributed to the study were as follows: Dr. Kate Armon, Mr. Joe Ellis‐Gage, Ms Holly Roper, Ms Vanja Briggs, and Ms Joanna Watts (Norfolk and Norwich University Hospitals); Dr. Liza McCann, Mr. Ian Roberts, Dr. Eileen Baildam, Ms Louise Hanna, Ms Olivia Lloyd, and Ms Susan Wadeson (The Royal Liverpool Children's Hospital, Alder Hey, Liverpool); Dr. Phil Riley and Ms Ann McGovern (Royal Manchester Children's Hospital, Manchester); Dr. Clive Ryder, Mrs. Janis Scott, Mrs. Beverley Thomas, Professor Taunton Southwood, and Dr. Eslam Al‐Abadi (Birmingham Children's Hospital, Birmingham); Dr. Sue Wyatt, Mrs. Gillian Jackson, Dr. Tania Amin, Dr. Mark Wood, Dr. Vanessa VanRooyen, and Ms Deborah Burton (Leeds General Infirmary, Leeds); Dr. Joyce Davidson, Dr. Janet Gardner‐Medwin, Dr. Neil Martin, Ms Sue Ferguson, Ms Liz Waxman, and Mr. Michael Browne (The Royal Hospital for Sick Children, Yorkhill, Glasgow); Dr. Mark Friswell, Professor Helen Foster, Mrs. Alison Swift, Dr. Sharmila Jandial, Ms Vicky Stevenson, Ms Debbie Wade, Dr. Ethan Sen, Dr. Eve Smith, Ms Lisa Qiao, Mr. Stuart Watson, and Ms Claire Duong (Great North Children's Hospital, Newcastle); Dr. Helen Venning, Dr. Rangaraj Satyapal, Mrs. Elizabeth Stretton, Ms Mary Jordan, Dr. Ellen Mosley, Ms Anna Frost, Ms Lindsay Crate, Dr. Kishore Warrier, and Ms Stefanie Stafford (Queens Medical Centre, Nottingham); Dr. Nathan Hasson, Mrs. Sue Maillard, Ms Elizabeth Halkon, Ms Virginia Brown, Ms Audrey Juggins, Dr. Sally Smith, Mrs. Sian Lunt, Ms Elli Enayat, Miss Laura Kassoumeri, Miss Laura Beard, Mrs. Yvonne Glackin, Dr. Beverley Almeida, Dr. Raquel Marques, Ms Stefanie Dowle, and Ms Charis Papadopoulou (Great Ormond Street Hospital, London); Dr. Kevin Murray (Princess Margaret Hospital, Perth, Western Australia); Dr. John Ioannou and Ms Linda Suffield (University College London Hospital, London); Dr. Muthana Al‐Obaidi, Ms Helen Lee, Ms Sam Leach, Ms Helen Smith, Dr. Anne‐Marie McMahon, Ms Heather Chisem, and Ms Ruth Kingshott (Sheffield's Children's Hospital, Sheffield); Dr. Nick Wilkinson, Ms Emma Inness, Ms Eunice Kendall, Mr. David Mayers, Ms Ruth Etherton, and Dr. Kathryn Bailey (Oxford University Hospitals, Oxford); Dr. Jacqui Clinch, Ms Natalie Fineman, and Ms Helen Pluess‐Hall (Bristol Royal Hospital for Children, Bristol); Ms Lindsay Vallance (Royal Aberdeen Children's Hospital); Ms Louise Akeroyd (Bradford Teaching Hospitals); Dr. Alice Leahy, Ms Amy Collier, Ms Rebecca Cutts, Dr. Hans De Graaf, Dr. Brian Davidson, Ms Sarah Hartfree, and Mr. Danny Pratt (University Hospital Southampton).
