# Supplementary material for: Muscle Biopsy Findings in Combination With Myositis‐Specific Autoantibodies Aid Prediction of Outcomes in Juvenile Dermatomyositis
Source: Arthritis Rheumatol. 2016 Oct 9;68(11):2806–16. doi: 10.1002/art.39753 (PMC5091622; doi:10.1002/art.39753)
Supplement: Supplementary file 1 — Supplementary Figure 1 [file ART-68-2806-s001.doc]

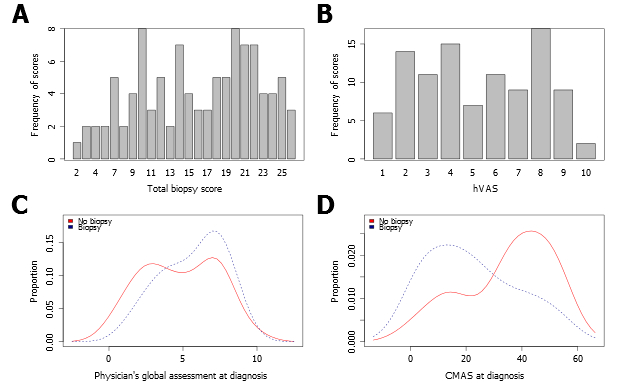


**Supplementary Figure 1. Distribution of biopsy scores within patients who underwent muscle biopsy, and distribution of clinical scores within all JDCBS patients. (A)** Total biopsy score of the 101 cases analyzed ranged from 2 to 26, with median 17 and interquartile range 10 to 21. **(B)** hVAS ranged from 1 to 10, with median 5 and interquartile range 3 to 7.75. Density plots for comparing the distribution of **(C)** physician’s global assessment at diagnosis and **(D)** CMAS at diagnosis, according to whether or not patients underwent biopsy, for all JDCBS patients. For the comparisons in (C) and (D), the 149 patients who underwent biopsy were compared to the 345 patients who did not. The additional biopsy samples not included in this analysis were either of insufficient quality to include or unavailable at the time of analysis. CMAS, Childhood Myositis Assessment Scale; hVAS, histopathologist’s visual analog scale global pathology score.
