# Supplementary material for: Muscle Biopsy Findings in Combination With Myositis‐Specific Autoantibodies Aid Prediction of Outcomes in Juvenile Dermatomyositis
Source: Arthritis Rheumatol. 2016 Oct 9;68(11):2806–16. doi: 10.1002/art.39753 (PMC5091622; doi:10.1002/art.39753)
Supplement: Supplementary file 2 — Supplementary Tables 1 [file ART-68-2806-s002.docx]

**Supplementary Table 1. Comparison of demographic and clinical features in JDCBS^a^ patients who underwent muscle biopsy versus those who did not undergo biopsy**

|  | Biopsy | No biopsy | | P-value^b^ |
| --- | --- | --- | --- | --- |
| Sex, (%)  Male  Female | 34.2%  65.8% | 27.6%  72.4% | 0.163 | |
| Ethnicity, (%)  White  Black  South Asian  Other | 68.2%  12.8%  7.4%  11.5% | 81.9%  7.1%  5.8%  5.3% | | 0.006 |
| Age of disease onset (years), median [IQR] | 6.8 [4.1 – 10.0] | 6.7 [4.0 – 10.1] | | 0.913 |
| Physician’s global assessment, median [IQR^c^] | 6.2 [4.0 – 7.3] | 4.9 [2.6 – 7.0] | | 0.042 |
| Childhood Myositis Assessment Scale, median [IQR] | 19 [8 – 33.8] | 38 [21 – 48] | | < 0.001 |
| Manual Muscle Testing and a Subset of Eight Muscles, median [IQR] | 43 [30.5 – 57.8] | 66 [52 – 78] | | < 0.001 |

^a^JDCBS, UK juvenile dermatomyositis cohort and biomarker study. For these comparisons, the 149 patients who underwent biopsy were compared to the 345 patients who did not undergo biopsy. The additional biopsy samples not included in this analysis were either of insufficient quality to include or unavailable at the time of analysis.

^b^Distributions of numeric variables between JDCBS patients who underwent biopsy and JDCBS patients who did not undergo biopsy, analyzed using Mann-Whitney test. Distributions of categorical variables between groups were analyzed using Fisher’s exact test.

^c^IQR, interquartile range

**Supplementary Table 2. Analysis of distribution of medications ever received across myositis-specific autoantibody cases and no-detectable autoantibody cases**

| Medication | Fisher’s exact p-value^a^ |
| --- | --- |
| Oral steroids | 1 |
| Intravenous steroids | 0.07785 |
| Methotrexate | 1 |
| Azathioprine | 0.2422 |
| Cyclophosphamide | 0.7828 |
| Hydroxychloroquine | 0.05706 |
| Intravenous immunoglobulin | 0.2101 |
| Mycophenolate mofetil | 1 |
| Infliximab | 0.4091 |
| Adalimumab | 1 |

^a^Fisher’s exact p-value for testing the null hypothesis that medications ever received are not distributed differently across myositis-specific autoantibody sub-groups. P-values below 0.005 were considered statistically significant.
